# Three-Layered Composite Scintillator Based on the Epitaxial Structures of YAG and LuAG Garnets Doped with Ce^3+^ and Sc^3+^ Impurities

**DOI:** 10.3390/ma17164025

**Published:** 2024-08-13

**Authors:** Sandra Witkiewicz-Łukaszek, Vitalii Gorbenko, Tetiana Zorenko, Jan Pejchal, Jiri A. Mares, Romana Kucerkova, Alena Beitlerova, Martin Nikl, Oleg Sidletskiy, Janusz Winiecki, Carmelo D’Ambrosio, Yuriy Zorenko

**Affiliations:** 1 Faculty of Physics, Kazimierz Wielki University in Bydgoszcz, Powstańców Wielkopolskich Street 2, 85-090 Bydgoszcz, Poland; 2 Institute of Physics, Academy of Sciences of the Czech Republic, 6253 Prague, Czech Republic; pejchal@fzu.cz (J.P.);; 3 Institute of Scintillation Materials, National Academy of Sciences of Ukraine, Av. Nauki 60, 61178 Kharkiv, Ukraine; sidletskiy@isma.kharkov.ua; 4 Prof. Franciszek Łukaszyk Oncology Center, Medical Physics Department, Dr Izabeli Romanowskiej Street 2, 85-796 Bydgoszcz, Poland; 5 Department of Oncology and Brachytherapy, Collegium Medicum in Bydgoszcz Nicholas Copernicus University in Toruń, Jagiellońska Street 13/15, 85-067 Bydgoszcz, Poland; 6 European Organization for Nuclear Research (CERN), Avenue des Particullers, EP-LHB Group, 1211 Geneva, Switzerland

**Keywords:** composite scintillators, liquid phase epitaxy, crystal, films, garnets, Ce and Sc dopants

## Abstract

In this study, we propose novel three-layer composite scintillators designed for the simultaneous detection of different ionizing radiation components. These scintillators are based on epitaxial structures of LuAG and YAG garnets, doped with Ce^3+^ and Sc^3+^ ions. Samples of these composite scintillators, containing YAG:Ce and LuAG:Ce single crystalline films with different thicknesses and LuAG:Sc single crystal substrates, were grown using the liquid phase epitaxy method from melt solutions based on PbO-B_2_O_3_ fluxes. The scintillation properties of the proposed composites, YAG:Ce film/LuAG:Sc film/LuAG:Ce crystal and YAG:Ce film/LuAG:Ce film/LuAG:Sc crystal, were investigated under excitation by radiation with α-particles from a ^239^Pu source, β-particles from ^90^Sr sources and γ-rays from a ^137^Cs source. Considering the properties of the mentioned composite scintillators, special attention was paid to the ability of simultaneous separation of the different components of mixed ionizing radiation containing the mentioned particles and quanta using scintillation decay kinetics. The differences in scintillation decay curves under α- and β-particle and γ-ray excitations were characterized using figure of merit (FOM) values at various scintillation decay intensity levels (1/e, 0.1, 0.05, 0.01).

## 1. Introduction

In recent years, the liquid phase epitaxy (LPE) method has been successfully used to create composite scintillators of the foswich type [1,2], based on the epitaxial structures of different oxide compounds containing single crystalline film (SCF) scintillators and bulk single crystal (SC) substrate scintillators for simultaneous registration of the different components of ionizing radiation (α- and β-particles and γ-quanta) [3,4,5,6,7]. Our recent works on the development of two-layer composite scintillators show the great potential of using garnet epitaxial structures for effective detection of mixed ionization fluxes, consisting of α-particles and γ-quanta, by separating the scintillation decay kinetics from the films and crystal parts of composite scintillators [3,4,5,6,7]. The multilayered epitaxial structures of complex oxide compounds (garners, perovskites and orthosilicates) are also very good promoting materials for creating composite scintillation screens [8,9], enabling developments of microtomographic devices with improved contrast and spatial resolution in comparison with single-layer screens [10,11].

**Figure 1:**
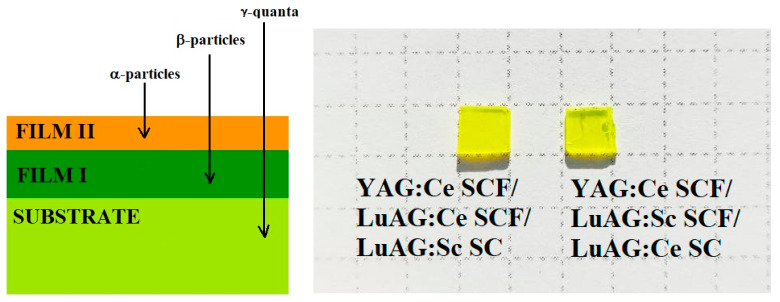
**Left**—scheme of three-layered composite scintillator for simultaneous registration of α- and β-particles and γ-rays. **Right**—three-layered composite scintillators YAG:Ce SCF_2_/LuAG:Ce SCF_1_/LuAG:Sc SC (**left**) and YAG:Ce SCF_2_/LuAG:Sc SCF_1_/LuAG:Ce SC (**right**).

The operation of the composite scintillator involves different interactions of various types of ionizing radiation sources with their SCF and the substrate parts. SCF scintillators composed of relatively “light” materials, like Y_3_Al_5_O_12_ (YAG) garnet [10,11,12], are used to register α- and β-particles with small penetration depths. The LPE method for manufacturing composite scintillators allows for producing film scintillators with thicknesses closely matching the penetration depth of the particles being detected. Specifically, the thickness required for the complete absorption of α-particles from ^239^Pu (5.15 MeV) and ^241^Am (5.5 MeV) sources is typically 12–15 μm [3,6,7]. On the contrary, the substrates produced from high-density materials, namely Ce^3+^-doped Lu_3_Al_5_O_12_ (LuAG) [13,14] and Gd_3_Ga_3_Al_2_O_12_ (GAGG) garnets [15,16,17], are dedicated to the registration of X- or γ-rays with high permeability to the interior of the materials (Figure 1). Notably, these crystals show a relatively high light yield of up to 25 and 50 photons per keV, and energy resolutions at 662 keV up to 8% and 4.5%, respectively, and are among the best scintillators for radiation monitoring and medical applications [13,14,15]. Among the latter applications, it is worth mentioning CT and PET diagnostics, as well as the possibility of using LuAG:Ce and GAGG:Ce scintillators for in situ measurement of radiation doses in brachytherapy procedures with ^192^Ir or other γ-ray sources. Moreover, the composite scintillators based on YAG, LuAG and GAGG garnets are good candidates for the development of composite detectors for measuring the alpha radiation doses of components of α-particles, ^7^Li ions and γ-quanta in the quantum range in boron-capture-neutron therapy (BNCT). 

To evaluate appropriate materials for a composite scintillator in terms of their ability to detect various components of mixed ionizing radiation, it is important to choose materials with appropriate scintillation properties. Of the most importance for this are the density (ρ), the effective atomic number (Z_eff_) and the amount of light absorbed per thickness of the material (absorption coefficient μ = ρZ^4^). Recently, for the creation two-layered composite scintillator materials, we have used well-known heavy scintillators based on LuAG garnet characterized by a high density of ρ = 6.73 g/cm^3^, an effective atomic number of Z_eff_ = 58.9 and an absorption coefficient of μ = 81 × 10^6^ g/cm^3^ [6]. As activators that can effectively emit in LuAG and YAG hosts with different wavelengths and scintillation decay kinetics, Pr^3+^, Sc^3+^ and Ce^3+^ ions were considered [3,6,18,19,20]. Specifically, the emission bands of LuAG:Ce, LuAG:Pr and LuAG:Sc compounds under high-energy excitation are located at 515 nm, 310 nm and 275–330 nm, respectively. The corresponding decay times of the main luminescence component for Ce^3+^, Pr^3+^ and Sc^3+^ dopants in the LuAG host are 50–58 ns, 19–28 ns and 245–610 ns, respectively, depending on the concentration of the dopants [6,18,19,20]. Based on the mentioned doped LuAG compounds, the two-layered LuAG:Sc SCF/LuAG:Ce SC and LuAG:Ce SCF/LuAG:Sc SC composite scintillators were created [8,9,16]. In addition, for registration of α-particles with small penetration depths, the relatively light YAG:Ce garnet, with the quite low density of ρ = 4.56 g/cm^3^, effective atomic number of Z_eff_ = 29 and absorption coefficient of μ = 3.8 × 10^6^ g/cm^3^, can be used as well [3,6,21,22,23].

In our previous works ([6], and references therein) we have shown that in LuAG:Ce SCF/LuAG:Sc SC and LuAG:Sc SCF/LuAG:Ce SC composite scintillators, the signal coming from the SCF and SC components can be separated with a large enough t_α_/t_γ_ decay time ratio (>1.5) in the quite narrow range of scintillation intensity decay from the 0.1 level down to the 0.01 level in the whole time interval. The results of our previous investigation also enable using the developed two-layered structures to produce three-layered composite scintillators for detection of different components of ionizing radiation (α- and β-particles and γ-rays) by means of the registration of the difference in the scintillation signals (pulse height spectra and decay kinetics) coming correspondingly from the SCF and SC parts of composite scintillators. 

In this paper, we present the results of the development of two novel three-layered YAG:Ce SCF_2_/LuAG:Ce SCF_1_/LuAG:Sc SC and YAG:Ce SCF_2_/LuAG:Sc SCF_1_ /LuAG:Ce SC composite scintillators prepared using the LPE method for simultaneous detection of α- and β-particles and γ-rays. This study represents a significant advancement in the ongoing search for innovative composite scintillators, aimed at detecting various components of mixed ionizing radiation and enhancing their utility in medical applications. During development of these new scintillators and optimization of their properties, we aim to improve the efficiency and accuracy of radiation detection, which is crucial for both diagnostic and therapeutic procedures in the medical field. This research builds on previous efforts and marks a crucial step forward in achieving more effective and versatile scintillation materials. 

## 2. Samples and Equipment

For creation of three-layered composite scintillators, samples of recently developed two-layered LuAG:Sc SCF_1_/LuAG:Ce SC and LuAG:Ce SCF_1_/LuAG:Sc SC composite scintillators [7,8] were used. Two new types of three-layered composite scintillators were grown using super-cooled melt solutions using a PbO-B_2_O_3_ flux by the Chairs of Optoelectronic Materials of the Physical Faculty of Kazimierz Wielki University in Bydgoszcz, Poland (COM FF UKW; see address^1^). Figure 1 shows the samples of the YAG:Ce SCF_2_/LuAG:Sc SCF_1_/LuAG:Ce SC and YAG:Ce SCF_2_/LuAG:Ce SCF_1_/LuAG:Sc SC composite scintillators. The growth conditions (temperature of growth and growth rate), thickness of the films and crystal parts of these three-layered epitaxial structures as well as their relative light yield (LY) are presented in Table 1. For comparison, the growth conditions, thickness and relative LY of two-layered LuAG:Sc SCF_1_/LuAG:Ce SC and LuAG:Ce SCF_1_/LuAG:Sc SC epitaxial structures were also added to Table 1. 

The size of the three-layered composite scintillators was around 5 × 5 × 0.5 mm, and they have quite irregular shapes resulting from cutting the larger LuAG:Sc SCF_1_/LuAG:Ce SC and LuAG:Ce SCF_1_/LuAG:Sc SC structures with 10 × 10 × 0.5 mm dimensions into four smaller pieces (Figure 1). The thickness of the films was measured using the weight method and calculated as h = (M − m)/ρ*S, where M is the mass of SCF and the substrate, m is the mass of the substrate, ρ is the density of the material and S is the surface of the substrate. The thicknesses of SCF_1_ and SCF_2_ were in the 10.5–21 μm range (Table 1).

The data presented in Table 1 indicate that the relative light yield (LY) of the upper parts of the two-layered epitaxial structures YAG:Ce SCF_2_/LuAG:Sc SCF_1_/LuAG:Ce SC and YAG:Ce SCF_2_/LuAG:Ce SCF_1_/LuAG:Sc SC exhibited relatively high values. Specifically, the relative LY was 54% for LuAG:Sc SCF_1_ samples and 126% for LuAG:Ce SCF_1_ samples. Moreover, the relative LY values of the YAG:Ce SCF upper parts of the three-layered composite scintillators were equal to 95% and 106%, respectively, compared to a standard YAG:Ce SCF sample with a known LY of 2600 ph/MeV under excitation by α-particles from a ^239^Pu (5.15 MeV) source (Table 1). The deviation in the LY of SCF_1_ and SCF_2_ scintillators depends on the content of the film material, the type of dopant and temperature of SCF growth, resulting in different activator/Pb flux dopant ratios in SCF content. Typically, such ratios increase with increasing temperature of growth and vice versa [6,7].

## 3. Experimental Technique

To characterize the luminescent and scintillation properties of SCF and bulk crystal parts in epitaxial structures of three-layered composite scintillators, we used absorption spectra, cathodoluminescence (CL) and radioluminescence (RL) spectra and scintillation decay kinetics under α- and β-particle and γ-ray excitations. These measurements were conducted at both COM FF UKW (see address^1^) and the Institute of Physics, Academy of Sciences in Prague, Czech Republic (FZU) (see address^2^). The absorption spectra were measured at COM FF UKW using a Jasco 760 UV–Vis spectrometer, covering the range of 200–1100 nm. Cathodoluminescence (CL) spectra were obtained at COM FF UKW with a JEOL JSM-820 electron microscope, Europe SAS (operating at U = 30 kV, I = 0.1 μA) equipped with a Stellar Net spectrometer and a TE-cooled CCD detector, which functions in the 200–925 nm range. The scintillation light yield (LY) was assessed using a setup comprising a Hamamatsu H6521 photomultiplier (PMP), Japan, a multichannel analyzer and a digital Tektronix TDS3052 oscilloscope, USA. The pulse height spectra, measured with a shaping time of 12 μs, were recorded under excitation by α-particles from a 239 Pu (5.15 MeV) source at COM FF UKW. These spectra were compared against a standard YAG: Ce SCF sample with a photoelectron yield of 360 ph/MeV and an LY of 2650 ph/MeV, as well as with reference LuAG:Ce and LuAG:Sc SC substrates produced by FZU and ISMA (Kharkiv, Ukraine).

More detailed investigations of the PHS under various shaping times (from 0.5 to 10 µs) were also carried out at the FZU, where we used the hybrid photomultiplier (HPMT) Photonis PPO475C, the spectroscopy amplifier Ortec 672, USA the Ortec 627, USA multichannel analyzer and a control PC. A setup with HPMT Photonis PPO470 was also used at CERN (see address) for PHS measurements with various X- and γ-energy lines and using a special α-particle ^241^Am source. At FZU, we also studied scintillation decay kinetics with α- (^239^Pu) and β- (^90^Sr) particles and γ-quanta (^137^Cs). For these measurements, we used a Hamamatsu R375 photomultiplier PMT, Japan and Textronix TOS 3052 oscilloscope, USA.

### 3.1. Absorption Spectra

**Figure 2:**
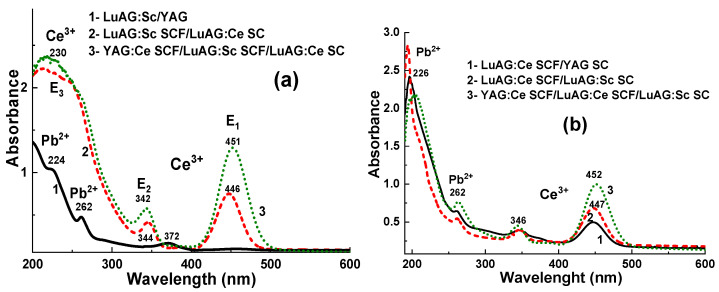
(**a**) Scheme of three-layered composite scintillator for simultaneous registration of α- and β-particles and γ-rays. (**b**) Three-layered composite scintillators YAG:Ce SCF_2_/LuAG:Ce SCF_1_/LuAG:Sc SC (**a**) and YAG:Ce SCF_2_/LuAG:Sc SCF_1_/LuAG:Ce SC (**b**).

The RT absorption spectra of the LuAG:Ce substrates, two-layered LuAG:Sc SCF_1_/LuAG:Ce SC and three-layered YAG:Ce SCF_2_/LuAG:Sc SCF_1_/LuAG:Ce SC epitaxial structures are shown in Figure 2a in comparison with the spectra of the LuAG:Sc SC substrates, two-layered LuAG:Ce SCF_1_/LuAG:Sc SC and three-layered YAG:Ce SCF_2_/LuAG:Ce SCF_1_/LuAG:Sc SC epitaxial structures (Figure 2b). For both types of composite scintillators (Figure 2), the absorption bands E_1_ at 446–452 nm and E_2_ at 342–346 nm are related to the 4f-5d (^2^E) transitions of Ce^3+^ ions in the LuAG:Ce SC substrate or LuAG:Ce SCF parts. Other Ce^3+^ absorption bands (E_3_) in these scintillators are located in the UV range approximately at 230 nm and related to the 4f-5d (T_2g_) transitions. Meanwhile, in the case of two-layered composition scintillators, the contribution of absorption of the additional YAG:Ce SCF2 layer results in the visible blue and red shifts at the positions of the E_2_ and E_1_ bands, respectively, due to the larger crystal field strength in the dodecahedral position of the YAG host in comparison with the LuAG matrix [24,25].

It is also worth noting here that the absorption spectra of all YAG:Ce and LuAG:Sc SCF scintillators grown from a PbO-based flux also possess two wide bands peaking around 262 nm and below 225 nm (Figure 2a,b). These bands are related to the ^1^S_0_→^3^P_1_ and ^1^S_0_→^1^P_1_ transitions, respectively, of Pb^2+^ flux-related impurity in the SCF samples [26,27]. The intensity of these bands strongly depends on the lead concentration in the film scintillators, which is inversely proportional to the temperature of SCF growth (see [26,27] for details).

### 3.2. Cathodoluminescence (CL) and Radioluminescence (RL) Spectra

**Figure 3:**
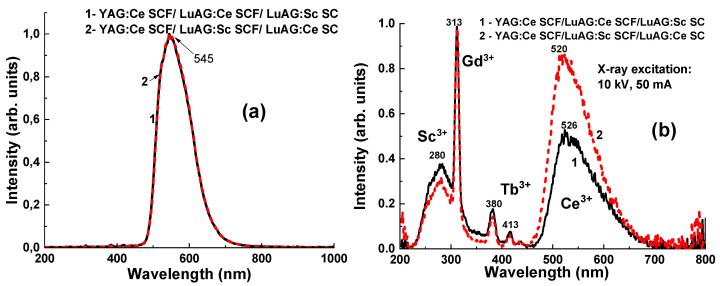
Normalized CL (**a**) and RL (**b**) spectra of YAG:Ce SCF_2_/LuAG:Ce SCF_1_/LuAG:Sc SC and YAG:Ce SCF_2_/LuAG:Sc SCF_1_/LuAG:Ce SC.

The CL and RL spectra of the YAG:Ce SCF_2_/LuAG:Ce SCF_1_/LuAG:Sc SC and YAG:Ce SCF_2_/LuAG:Sc SCF_1_/LuAG:Ce SC composite scintillators are presented in Figure 3a,b, respectively. Due to the low penetration depth of electrons in the material (around 1 μm), the CL spectra of the composite scintillators are determined by the luminescence of their YAG:Ce SCF parts. Specifically, the CL spectra of YAG:Ce SCFs in both types of composite scintillators are characterized by a dominant emission band with a maximum at 545 nm assigned to the 5d–4f transitions of Ce^3+^ ions. 

The RL spectra of the YAG:Ce SCF1/LuAG:Ce SCF2/LuAG:Sc SC and YAG:Ce SCF_2_/LuAG:Sc SCF_2_/LuAG:Ce SC structures (Figure 3b) under X-ray excitation (Cu_Kα_; 10 kV, 50 mA) show wide emission bands with maxima at 520 and 526 nm, which is typical for Ce^3+^ 5d-4f emission. It is worth noting here that the maxima of Ce^3+^ emission bands in the RL spectra for both composites are significantly blue-shifted in comparison with the CL spectra caused by YAG:Ce SCF luminescence. Considering that the Ce^3+^ emission band in the LuAG garnet peaked at 515 nm [24,25], the blue shift in the RL spectra with respect to the CL spectra in both types of composite scintillators is due to the dominant contribution of the luminescence of LuAG:Ce SCF and LuAG:Ce SC in these RL spectra. This also means that X-rays are significantly absorbed by LuAG:Ce SCF and LuAG:Ce SC in the first and second types of composite scintillators due to the high density and effective absorption number of the LuAG host. Indeed, the intensity of the Ce^3+^-related emission band and the blue shift rate are much higher in the YAG:Ce SCF_2_/LuAG:Sc SCF_1_/LuAG:Ce SC composite than those in the YAG:Ce SCF_2_/LuAG:Ce SCF_1_/LuAG:Sc SC counterpart due to the much greater thickness of the LuAG:Ce SC substrate (500 µm) compared to LuAG:Ce SCF_1_ (14 µm). 

The broad complex emission band in the UV range (220–450 nm) peaking around 280 nm in the RL spectra of both composites is typical for the intrinsic luminescence of YAG SC grown from a melt [27,28,29,30,31,32]. The nature of this band is caused by the emission of excitons localized to and bound with Y_Al_ antisite defects in the YAG SC substrate [28,33,34,35]. The sharp lines at 313 nm and 380 nm and 413 nm are caused by the luminescence of Gd^3+^ and Tb^3+^ trace impurities in the row materials used for the preparation of the YAG substrate [22,23,24,25,26,27,28,33,34,35,36,37]. 

### 3.3. Scintillation Properties of Composite Scintillators

#### 3.3.1. Pulse Height Spectra (PHS)

**Figure 4:**
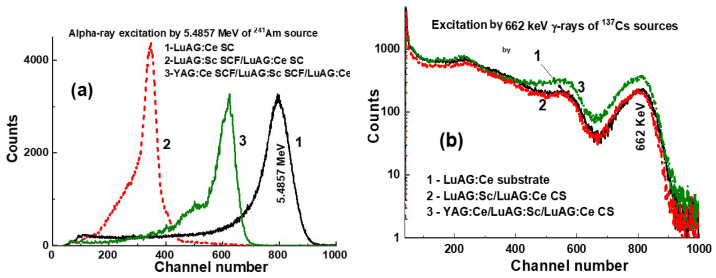
PHS of LuAG:Ce SC substrate (1) and two-layered LuAG:Sc SCF_1_/LuAG:Ce SC structure (2), as well as three-layered YAG:Ce SCF_2_/LuAG:Sc SCF_1_/LuAG:Ce SC composite scintillators (3), were measured with a shaping time of 3 µs. Measurements were conducted under α-particle excitation with an energy of 5.4857 MeV by a ^241^Am source (**a**) and under γ-ray excitation with an energy of 662 keV by a ^137^Cs source (**b**).

The PHS of the LuAG:Ce SC substrate, two-layered LuAG:Sc SCF1/LuAG:Ce SC structure and three-layered YAG:Ce SCF_2_/LuAG:Sc SCF1/LuAG:Ce SC composite scintillators under excitation or α-particles from a ^241^Am source and γ-rays from a ^137^Cs source, measured with a 3 μs shaping time, are presented in Figure 4a,b, respectively. 

The main peaks in Figure 4a correspond to the total energy absorption of α-rays with an energy of 5.4857 MeV. It is worth noting that the positions of the main peaks, observed in Figure 4a, are substantially different for YAG:Ce SCF_2_ and LuAG:Sc SCF_1_ for both composite scintillators and for the LuAG:Ce SC substrate. This means that α-particles excite only the SCF parts of composite scintillators. Figure 4a shows also that the LY values of the YAG:Ce SCF_2_ and LuAG:Sc SCF_1_ parts of composite scintillators under excitation by α-particles are smaller by 1.3 and 2.3 times, respectively, in comparison with the LY value for the LuAG:Ce substrate. These results for the LY of composite scintillators are consistent with the results obtained for the samples presented in Table 1 under excitation by a ^239^Pu source, measured with a shaping time of 12 μs. Such a lower LY of SCF samples is caused mainly by the negative influence of Pb flux-related impurity on the scintillation properties of SCFs of different oxide compounds grown from a PbO-based flux [38,39].

LuAG:Sc SCF_1_/LuAG:Ce SC and YAG:Ce SCF_2_/LuAG:Sc SCF_1_/LuAG:Ce SC composite scintillators are excited by γ-rays from a ^137^Cs source, with the primary peaks in the pulse height spectra (PHS) corresponding to the total absorption of γ radiation with an energy of 661.66 keV (Figure 4b). Additionally, a secondary peak is observed at a lower energy of 32 keV, which corresponds to the low-energy line of the ^137^Cs source. Notably, the main photopeaks in Figure 4b are located at similar positions for both the LuAG:Sc SCF_1_/LuAG:Ce SC and YAG:Ce SCF_2_/LuAG:Sc SCF_1_/LuAG:Ce composite scintillators and the LuAG:Ce substrate, and this means that γ-rays mainly excite the substrate.

**Figure 5:**
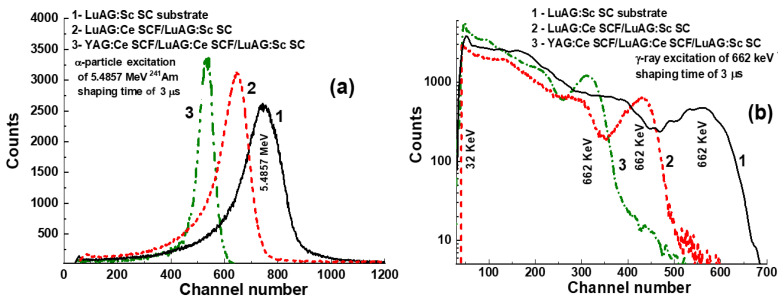
PHS of LuAG:Sc SC substrate (1), two-layered LuAG:Ce SCF_1_/LuAG:Sc SC structure (2) and three-layered YAG:Ce SCF_2_/LuAG:Ce SCF_1_/LuAG:Sc SC (3) composite scintillators were measured in a time range of 3 µs. Measurements were conducted under α-particle excitation with an energy of 5.4857 MeV by a ^241^Am source (**a**) and under γ-ray excitation from a ^137^Cs source with an energy of 662 keV (**b**).

The PHS of the LuAG:Sc SC substrate and doubly layered and triply layered LuAG:Ce SCF_1_/LuAG:Sc SC and YAG:Ce SCF_2_/LuAG:Sc SCF_1_/LuAG:Ce SC composite scintillators under α-particle excitation by the ^241^Am source and γ-ray excitation by the ^137^Cs source are presented in Figure 5a,b, respectively. It is noteworthy that at registration of α-particles, the main peaks are shifted relative to each other and to the substrate (Figure 5), because α-particles excite only the film part of composite scintillators. At registration of γ-rays, the main peaks are also shifted (Figure 5b), indicating the excitation of both the substrate and SCFs. Recently, a similar shift in the position of the 662 keV γ-ray peaks was confirmed in the case of double-layer LuAG:Ce SCF_1_/LuAG:Sc SC composite scintillators [6]. In this work, we also confirm that the total absorption of 662 keV γ-rays also depends on the type, thickness and LY SCF and crystal parts of the composite scintillator. For example, for the YAG:Ce SCF_2_/LuAG:Sc SCF_1_/LuAG:Ce SC composite scintillator, the LY of the LuAG:Ce substrate is so high that the influence of YAG:Ce SCF_2_ and LuAG:Sc SCF_1_ on the 662 keV positions of the γ-ray peak is negligible (Figure 4b). However, for the YAG:Ce SCF_2_/LuAG:Ce SCF_1_/LuAG:Sc SC composite scintillator, the LY of the LuAG:Sc substrate is not as high as that of the LuAG:Ce substrate, and the influence of the heavy and efficient LuAG:Ce SCF_1_ scintillator and the lightweight but efficient YAG:Ce SCF_2_ scintillator is significant, as seen in Figure 5b.

**Figure 6:**
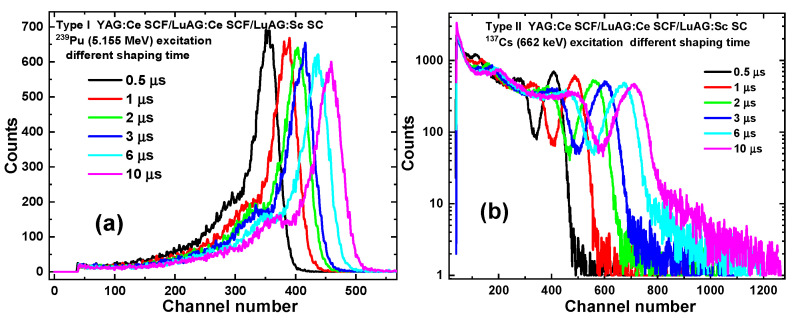
PHS of YAG:Ce SCF_2_/LuAG:Sc SCF_1_/LuAG:Ce SC composite scintillators measured with shaping time in the 0.5–10 µs range under α-particle excitation with an energy of 5.15 MeV from a ^239^Pu source (**a**) and under γ-ray excitation from a ^137^Cs source with an energy of 662 keV (**b**).

**Figure 7:**
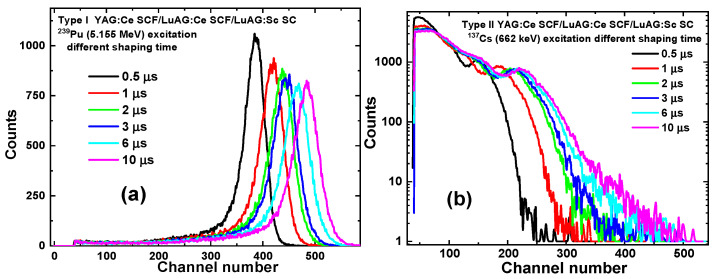
PHS of YAG:Ce SCF_2_/LuAG:Ce SCF_1_/LuAG:Sc SC composite scintillators measured with shaping time in the 0.5–10 µs range under α-particle excitation with an energy of 5.15 MeV from a ^239^Pu source (**a**) and under γ-ray excitation from a ^137^Cs source with an energy of 662 keV (**b**).

**Figure 8:**
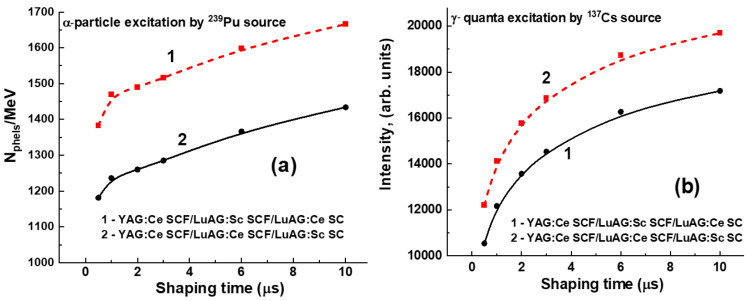
Dependence of the LY of the YAG:Ce SCF_2_/LuAG:Sc SCF_1_/LuAG:Sc SC (1) and YAG:Ce SCF_2_/LuAG:Ce SCF_1_/LuAG:Sc SC (2) composite scintillators under excitation by α-particles from a ^239^Pu source (**a**) and γ-rays from a ^137^Cs source (**b**).

Figure 6, Figure 7 and Figure 8 present the LY (in the photons per MeV) for YAG:Ce SCF_2_/LuAG:Sc SCF_1_/LuAG:Ce SC and YAG:Ce SCF_2_/LuAG:Ce SCF_1_/LuAG:Sc SC composite scintillators measured with different shaping times in the 0.5–10 μs range under α-particle excitation with a ^239^Pu source (a) and γ-ray excitations with a ^137^Cs source (b). For YAG:Ce SCF_2_/LuAG:Sc SCF_1_/LuAG:Ce SC and YAG:Ce SCF_2_/LuAG:Ce SCF_1_/LuAG:Sc SC composite scintillators excited by α-particles from a ^239^Pu source with an energy of 5.15 MeV, a similar trend of LY increase is observed. Specifically, the LY values increase from values of 1181 ph/MeV to 1434 ph/MeV (21.4%) and from 1383 ph/MeV to 1666 ph/MeV (20.5%), respectively, for the shaping time changes from 0.5 to 10 μs, respectively (see Table 2). On the contrary, we have observed notably different trends in the LY value changes under 661.66 keV γ-ray excitation for the YAG:Ce SCF_2_/LuAG:Sc SCF_1_/LuAG:Ce SC and YAG:Ce SCF_2_/LuAG:Ce SCF_1_/LuAG:Sc SC composite scintillators. Specifically, the LY values in these scintillators change from 12,210 to 19,695 ph/MeV (61%) and from 10,453 to 17,177 ph/MeV (64%), respectively, when the shaping times increase in the 0.5–10 µs interval.

#### 3.3.2. Scintillation Decay Kinetics

**Figure 9:**
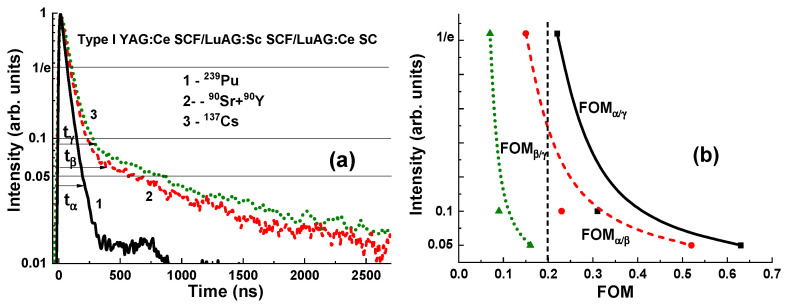
(**a**) Scintillation decay curves of YAG:Ce SCF_2_/LuAG:Sc SCF_1_/LuAG:Ce SC composite scintillators under excitation by α- and β-particles and γ-quanta. (**b**) FOM values under registration of the mentioned types of radiation.

**Figure 10:**
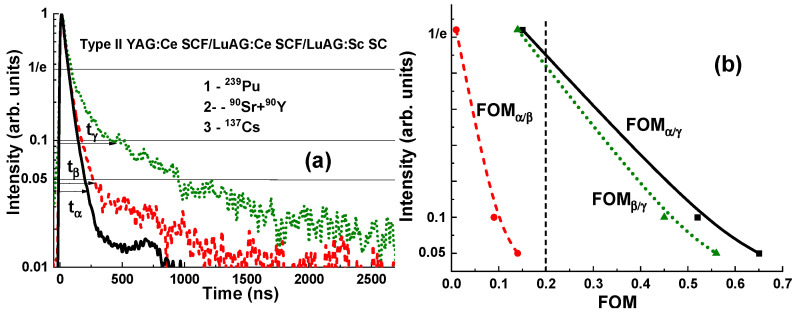
(**a**) Separation of the scintillation decay curves of YAG:Ce SCF_2_/LuAG:Ce SCF_1_/LuAG:Sc SC composite scintillators under excitation by α- and β-particles and γ-quanta. (**b**) FOM values of the mentioned composite scintillators under registration of the mentioned types of radiation.

Figure 9 and Figure 10 present the scintillation decay curves (a) and figure of merit (FOM) (b) values of the YAG:Ce SCF_2_/LuAG:Sc SCF_1_/LuAG:Ce SC and YAG:Ce SCF_2_/LuAG:Ce SCF_1_/LuAG:Sc SC composite scintillators under registration of α- (^239^Pu) and β- (^90^Sr) particle and γ-quantum (^137^Cs) radiations. Scintillating decay profiles from two types of SCF and SC components can be separated in the whole time interval from 0 to 3000 ns (Figure 9a and Figure 10a). We analyzed the scintillation decay kinetics to 1/e, 0.1 and 0.05 levels under the above-mentioned excitations. The differences in the scintillation decay times are presented using the FOM values ${FOM}_{\beta \gamma }=\left|\frac{\left(t_{\beta }-t_{\gamma }\right)}{\left(t_{\beta }+t_{\gamma }\right)}\right|$, ${FOM}_{\alpha \beta }=\left|\frac{\left(t_{\alpha }-t_{\beta }\right)}{\left(t_{\alpha }+t_{\beta }\right)}\right|$, ${FOM}_{\beta \gamma }=\left|\frac{\left(t_{\beta }-t_{\gamma }\right)}{\left(t_{\beta }+t_{\gamma }\right)}\right|$ and ${FOM}_{\alpha \gamma }=\left|\frac{\left(t_{\alpha }-t_{\gamma }\right)}{\left(t_{\alpha }+t_{\gamma }\right)}\right|$, where the differences in scintillation decay times t_α_, t_β_ and t_γ_ for the different levels (1/e, 0.1, 0.05) of the registration of the scintillation decay profiles were used to calculate the difference in scintillation decay times under simultaneous registration of the α-, β- and γ-excitations (Figure 9b and Figure 10b). All the data are collected in Table 3. In general, for effective separation of the two decay curves, the corresponding decay times at the selected decay intensity level must differ by at least 1.5 times. This means that the corresponding FOM values must be at least 0.2 or larger.

As can be seen from Figure 9a and Figure 10a, the scintillation response under α-particle excitation of the YAG:Ce SCF scintillators is significantly faster than that under β-particle and γ-quantum excitations of both types of composition scintillators under study. 

For the YAG:Ce SCF_2_/LuAG:Sc SCF_1_/LuAG:Ce SC composite scintillator, the largest separation occurs between the decay curves under α-particle and γ-ray excitations, and under β-particle and γ-ray excitations, where the largest FOMα/γ and FOMβ/γ ratios are equal to 0.63 and 0.52, respectively, at the 0.05 intensity levels (see Figure 9b and Table 3). However, for this type of composite scintillator, small FOM_β/γ_ values (0.07–0.16) were observed at the separation decay curves under simultaneous β-particle and γ-ray excitations (Figure 9b and Table 3). Therefore, for this type of scintillator, the FOM_β/γ_ values require improvement to at least a value of 0.2 by means of acceleration of the decay profile of the LuAG:Sc SCF_1_ scintillator. This can be accomplished by alloying the Ga to this SCF scintillator in a suitable concentration [29,40], doping with a double-charged Mg^2+^ dopant [30] or other coactivators [31] and/or increasing the thickness of the YAG:Ce SCF_2_ scintillators to at least 20–25 µm.

The differences between the scintillation decay curves in YAG:Ce SCF_2_/LuAG:Ce SCF_1_/LuAG:Sc SC under excitation by α-particles and γ-rays and under excitation by β-particles and γ-rays are also very high and equal to FOM_α_**_/_**_γ_ = 0.65 and FOM_β_**_/_**_γ_ = 0.56, respectively, at the 0.05 intensity levels. However, for this type of composite scintillator, a much smaller separation is observed between the decay curves under excitation by α- and β-particles, and the highest FOM_α_**_/_**_β_ value is equal to only 0.14 at the 0.05 intensity level. The improvement of the FOM_α_**_/_**_β_ value to at least 0.2 can be achieved by means of slowing the decay kinetics of the LuAG:Ce SCF_1_ scintillator using Gd^3+^ or Tb^3+^ doping of this SCF scintillator in a suitable concentration (see [6] for details). The second way to raise the FOM_α_**_/_**_β_ value is to increase the thickness of the LuAG:Ce SCF_1_ scintillators to at least 30–40 µm at the same thickness as their bulk SC part.

## 4. Conclusions

Two types of three-layered composite scintillators based on the YAG:Ce SCF_2_/LuAG:Sc SCF_1_/LuAG:Ce SC and YAG:Ce SCF_2_/LuAG:Ce SCF_1_/LuAG:Sc SC epitaxial structures were grown, using the LPE method, from melt solutions using a PbO-B_2_O_3_ flux. The luminescent and scintillation properties of the film and substrate parts of these composites were investigated using absorption, cathodoluminescent spectra, LY and scintillation decay kinetics under α- and β-particle and γ-ray excitations.

It has been found that both types of developed composite scintillators can effectively separate the scintillation signals originating from their two film layers and bulk parts when registering mixed radiation fluxes containing α- and β-particles and γ-rays. Specifically, the YAG:Ce SCF_2_/LuAG:Sc SCF_1_/LuAG:Ce SC and YAG:Ce SCF_2_/LuAG:Ce SCF_1_/LuAG:Sc SC epitaxial structures exhibit notable differences in scintillation decay kinetics at three intensity levels—1/e, 0.1 and 0.05—over a wide time interval of up to 3000 ns under excitation by α- and β-particles and γ-rays. Additionally, we have identified that the most effective method for simultaneous recording of mixed ionizing radiation is to measure it at a level of 5% of the peak scintillation decay intensity.

The differences in the scintillation decay kinetics of composite scintillators at registration of the different types of radiation can be characterized by their respective figure of merit (FOM) values. The largest FOM_α/γ_ value, equal to 0.63–0.65 for simultaneous α/γ registration, was obtained for both the YAG:Ce SCF_2_/LuAG:Sc SCF_1_/LuAG:Ce SC and YAG:Ce SCF_2_/LuAG:Ce SCF_1_/LuAG:Sc SC composite scintillators at a 0.05 scintillation decay level (Table 2). Meanwhile, for simultaneous α/β-particle registration, the FOM_α/β_ = 0.52 value is significantly larger in the YAG:Ce SCF_2_/LuAG:Sc SCF_1_/LuAG:Ce SC composition scintillator than the value of FOM_α/β_ = 0.14 for its YAG:Ce SCF_2_/LuAG:Ce SCF_2_/LuAG:Sc SC counterpart at the 0.05 decay level (see Table 2). 

However, the highest FOM_β/γ_ = 0.16 value for the YAG:Ce SCF_2_/LuAG:Sc SCF_1_/LuAG:Ce SC composite scintillator is not satisfactory for the effective separation of mixed β/γ excitation and requires improvement to at least a value of 0.2 by means of acceleration of the decay profile of the second LuAG:Sc SCF scintillator. This can be accomplished by Ga doping of this SCF scintillator in the appropriative concentration (Figure 4a), Mg^2+^ co-doping and/or increasing the thickness of the first YAG:Ce SCF scintillators to at least 20–25 µm. Similarly, the highest FOM_α/β_ = 0.14 value for the YAG:Ce SCF_2_/LuAG:Ce SCF_1_/LuAG:Sc SC scintillator is also not enough for the effective separation of mixed α/β excitation and requires improvement to a value above 0.2 by means of slowing of the decay profile of the LuAG:Ce SCF scintillator. This can be performed by Gd or Tb doping of the LuAG:Ce SCF scintillator in a suitable concentration [6] and/or increasing the thickness of this SCF scintillator to 30–40 µm. 

In conclusion, we are optimistic that by optimizing the composition, activator and co-dopant concentrations, thickness of SCF and substrate scintillators, and LPE growth conditions, the development of three-layered composite scintillators based on doped LuAG garnet [32,41,42] or other heavy scintillation compounds [43,44] will mark a significant advancement in the creation of next-generation multilayered scintillators. These optimized composite scintillators are expected to play a crucial role in the simultaneous registration of different components of mixed radiation fluxes. This progress could pave the way for more efficient and accurate detection methods, benefiting various applications in scientific research, medical diagnostics and radiation monitoring.

## Figures and Tables

**Table 1:** Growth conditions of composite scintillators and the relative LY of SCF parts of composite scintillators, under α-particle excitation by a ^239^Pu (5.15 MeV) source and measured with a shaping time of 12 μs, in comparison with the reference YAG:Ce SCF sample (100%) with a photoelectron LY of 360 ph/MeV (a LY of 2600 ph/MeV). * Relative LY of the upper SCF part of the composite scintillators.

Film II (Thickness)	Film I (Thickness)	Substrate (Thickness)	Temperature of Growth, °C	Growth Rate, μm/min	Relative LY, % *
-	LuAG:Sc (15 μm)	LuAG:Ce (500 μm)	980	0.21	54
-	LuAG:Ce (21 μm)	LuAG:Sc (500 μm)	985	0.2	126
YAG:Ce (15 μm)	LuAG:Sc (15 μm)	LuAG:Ce (500 μm)	982	0.16	95
YAG:Ce (10.5 μm)	LuAG:Ce (14 μm)	LuAG:Sc (500 μm)	985	0.2	106

**Table 2:** Scintillation characteristics of YAG:Ce SCF_2_/LuAG:Sc SCF_1_/LuAG:Ce SC and YAG:Ce SCF_2_/LuAG:Ce SCF_1_/LuAG:Sc SC composite scintillators: LY (Ph/MeV) and energy resolution ER (%), measured with the 0.5–10 μs shaping time, as well as variation in the LY, LY (10 μs)—LY (0.5 μs)/LY (0.5 μs), measured with the 0.5 and 10 μs shaping times.

Type	Content of Composite Scintillators	SCF_1_/SCF_2_/SC Substrate Thickness, μm	LY in 0.5–10 μs ^239^Pu/^137^Cs exc., ph/MeV	ER in 0.5–10 μs ^239^Pu/^137^Cs exc., %	Difference in LY 0.5–10 μs (%)
I	YAG:Ce SCF_2_/LuAG:Sc SCF_1_/LuAG:Ce SC	15/14/500	1181–1434 10,543–17,177	12.5–14.1 16.3–17.9	+21.4 +62.9
II	YAG:Ce SCF_2_/LuAG:Ce SCF_1_/LuAG:Sc SC	10.5/14/500	1383–1666 12,210–19,695	12.6–14.1 12.9–17.2	+20.5 +61.3

**Table 3:** Table of values of scintillation decay times under α- and β-particle and γ-ray excitations and FOM (figure of merit) values at different intensity levels (1/e, 0.1, 0.05).

**YAG:Ce SCF_2_/LuAG:Sc SCF_1_/LuAG:Ce SC**			
**Decay Time**	**α** **ex.**	**β** **ex.**	**γ** **ex.**
**t_1/e,_ ns**	68	93	106
**t_0.1,_ ns**	147	234	278
**t_0.05,_ ns**	193	616	845
**FOM of YAG:Ce SCF_2_/LuAG:Sc SCF_1_/LuAG:Ce SC**			
**Decay Level**	**FOM_α_** **_/γ_**	**FOM_α_** **_/β_**	**FOM_β_** **_/γ_**
**1/e**	0.22	0.15	0.07
**0.1**	0.31	0.23	0.09
**0.05**	0.63	0.52	0.16
**YAG:Ce SCF_2_/LuAG:Ce SCF_1_/LuAG:Sc SC**			
**Decay Time**	**α** **_ex_**	**β** **_ex_**	**γ** **_ex_**
**t_1/e,_ ns**	67.5	69.5	91.5
**t_0.1,_ ns**	146	175	467
**t_0.05,_ ns**	200	266	952
**FOM of YAG:Ce SCF_2_/LuAG:Ce SCF_1_/LuAG:Sc SC**			
**Decay Level**	**FOM_α_** **_/γ_**	**FOM_α_** **_/β_**	**FOM_β_** **_/γ_**
**1/e**	0.15	0.01	0.14
**0.1**	0.52	0.09	0.45
**0.05**	0.65	0.14	0.56

## Data Availability

The raw data supporting the conclusions of this article will be made available by the authors on request.
